# Using the Vitiligo Noticeability Scale in clinical trials: construct validity, interpretability, reliability and acceptability

**DOI:** 10.1111/bjd.21671

**Published:** 2022-07-07

**Authors:** Jonathan M. Batchelor, Sonia Gran, Paul Leighton, Laura Howells, Alan A. Montgomery, Wei Tan, Isma Ahmed, Kim S. Thomas

**Affiliations:** ^1^ Centre of Evidence Based Dermatology, School of Medicine University of Nottingham Nottingham UK; ^2^ Nottingham Clinical Trials Unit, School of Medicine University of Nottingham Nottingham UK; ^3^ Department of Dermatology King’s College Hospital NHS Foundation Trust, Beckenham Beacon Beckenham BR3 3QL UK

## Abstract

**Background:**

Validated outcome measures are needed for vitiligo trials.

**Objectives:**

To assess construct validity, interpretability, reliability and acceptability of the Vitiligo Noticeability Scale (VNS).

**Methods:**

We used images of vitiligo before and after treatment, plus outcome data, from the HI‐Light Vitiligo trial. We compared outcome assessments made by trial participants with assessments of images by clinicians and people with vitiligo who were not trial participants [Patient and Public Involvement (PPI) panel]. Hypothesis testing assessed psychometric properties of the VNS, with κ statistics to assess agreement between outcomes. Three focus groups and two online discussion groups provided insight into the use of VNS by people with vitiligo.

**Results:**

Our hypothesis of a positive association between VNS and participant‐reported global treatment success was supported for trial participants (κ = 0·41 if VNS success was defined as ≥ 4; κ = 0·71 if VNS success was defined as ≥ 3), but not for the blinded PPI panel (κ = 0·28). As hypothesized, the association with participant‐reported global success was higher for VNS (κ = 0·41) than for clinician‐reported percentage repigmentation (κ = 0·17). Seventy‐five per cent of trial participants valued a VNS of 3 (partial response) as a treatment success. Test–retest reliability was good: κ = 0·69 (95% confidence interval 0·63–0·74). Age and skin phototype did not influence interpretation of the VNS scores. To people with vitiligo, the VNS is an acceptable and meaningful patient‐reported outcome measure.

**Conclusions:**

Trial participants may assess their vitiligo differently compared with blinded assessors. A VNS score of 3 may be more highly valued by people undergoing vitiligo treatment than was previously thought.

**What is already known about this topic?**

- Vitiligo is a common condition, and can have a considerable psychological impact.
- A Vitiligo Core Outcome Set is being developed, to enable the results of vitiligo trials to be compared and combined more easily.
- The Vitiligo Noticeability Scale (VNS) is a patient‐reported outcome measure (PROM) developed in partnership with people with vitiligo; initial validation studies have been promising.

**What does this study add?**

- The VNS shows good construct validity, reliability and acceptability; it can be used in all ages and skin phototypes.
- All five levels of the VNS scale should be reported for transparency, to aid interpretation of trial findings, and to facilitate meta‐analysis in systematic reviews.
- VNS assessments made by trial participants and independent observers are likely to be qualitatively different, making blinded assessment of VNS by independent observers difficult to interpret.
- Blinding of participants to trial interventions is recommended whenever possible.

**What are the clinical implications of the work?**

- The VNS can be used as a PROM to assess the cosmetic acceptability of repigmentation at individual patches of vitiligo.
- A VNS score of 3 or more is likely to be valued by patients as a treatment success.

## Graphical abstract

• A Vitiligo Core Outcome Set is being developed, to enable the results of vitiligo trials to be compared and combined more easily • The VNS shows good construct validity, reliability and acceptability. It can be used in all ages and skin phototypes • The VNS can be used as a PROM to assess cosmetic acceptability of repigmentation at individual patches of vitiligo

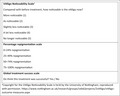

**Linked Comment:** A.G. Pandya and K. Ezzedine. Br J Dermatol 2022; 187:461–462.

Vitiligo is a condition causing patches of skin depigmentation.^1^ Systematic reviews have identified many vitiligo trials, but these often assess outcomes in different ways and use unvalidated outcome measures, preventing the combination of results in meta‐analyses and leading to research waste.^1^, ^2^, ^3^, ^4^

In response to the need for better‐quality patient‐reported outcome measures (PROMs) for vitiligo,^5^, ^6^ we developed the Vitiligo Noticeability Scale (VNS; Figure 1) in collaboration with patients with vitiligo and provisionally validated it for use as a PROM assessing ‘cosmetically acceptable repigmentation’ at a target patch of vitiligo in clinical trials.^7^, ^8^ Using data and images of treatment response following 9 months of treatment in the HI‐Light Vitiligo trial,^9^ we sought to further evaluate psychometric properties of the VNS, and conduct qualitative exploration of how the VNS is used and interpreted by people with vitiligo.

**Figure 1:**
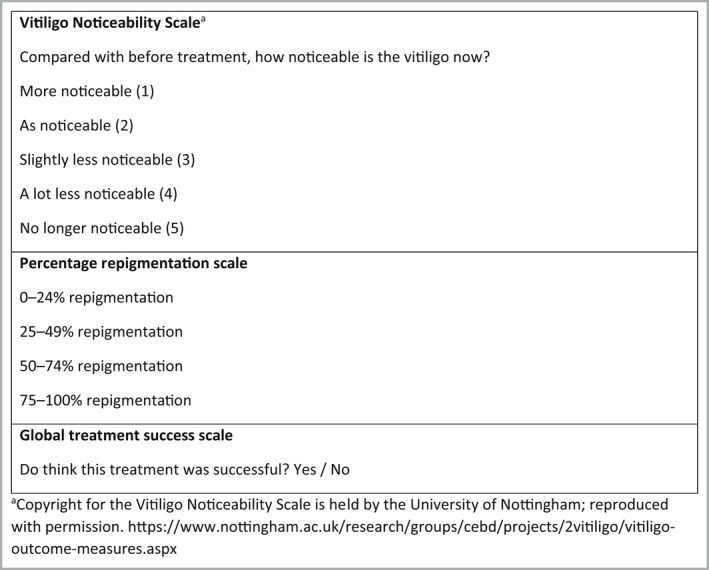
Scales used: Vitiligo Noticeability Scale; Percentage repigmentation scale; Global treatment success scale.

Our aim was to validate the VNS as a measure of cosmetic acceptability of repigmentation, to inform its use in clinical trials. The objectives were:

**1** to assess the construct validity, interpretability and reliability of the VNS, and its suitability for use in blinded assessment of digital images as an objective outcome measure in trials;

**2** to obtain qualitative insights into how people with vitiligo use the VNS to assess their vitiligo.

## Patients and methods

This study was conducted in accordance with COSMIN guidance.^10^, ^11^ Ethical approval was not required for secondary analysis of the HI‐Light trial dataset; this used digital image pairs from participants who had given informed consent for their trial data to be used in future research. For the focus groups, approval was obtained from the University of Nottingham Faculty of Medicine and Health Sciences Research Ethics Committee (Ref 387‐1909).

### Data source

Participants in the HI‐Light trial chose one vitiligo patch in which they most wanted to see an improvement after treatment.^9^ Digital images were taken of this patch at baseline and after 9 months of treatment. Trial participants completed the VNS at 9 months (comparing the patch on their skin with the baseline image) and assessed the vitiligo using a ‘global treatment success’ scale (Figure 1).

In addition, a panel of three people with vitiligo who were not trial participants [the Patient and Public Involvement (PPI) panel] rated the before and after image pairs using the VNS and global treatment success scale. A subsample of the image pairs was assessed on two occasions by an additional 10 PPI raters (13 in total) to assess the test–retest reliability of the VNS.

Three clinicians, blinded to trial treatment allocation, assessed all image pairs and rated them for global treatment success and percentage repigmentation (a scale used in most vitiligo trials; Figure 1).

### Psychometric properties explored

#### Construct validity

Construct validity is the degree to which scores on a measurement instrument are consistent with hypotheses that predefine the relationship between that instrument and other relevant scales.^12^ We took participant‐rated global treatment success to be our main comparator of interest, because the overall assessment of success by the person receiving treatment is the most important construct.

We hypothesized that:

**1** there would be a positive association between VNS scores (treatment success ≥ 4 on the VNS) and participant‐reported global treatment success at end of treatment (9 months), with κ ≥ 0·4;

**2** that VNS scores would have better association with participant‐reported global treatment success than percentage repigmentation at the end of treatment.

These hypotheses are consistent with those used when initially validating the VNS.^8^

#### Interpretability

The interpretability of a measurement instrument refers to ‘the degree to which one can assign qualitative meaning … to an instrument’s quantitative scores or change in scores’.^10^ Our previous work suggested that a score of ≥ 4 on the five‐point VNS represents treatment success, and 3 represents partial success.^8^

We used an anchor‐based approach^12^ to evaluate the interpretability of the VNS, using ‘participant‐reported global treatment success’ (Figure 1) as an anchor. Based on our previous work,^8^ we considered a VNS score of 4 or 5 to represent treatment success (in terms of cosmetic acceptability of repigmentation) if at least 85% of the images allocated to that score were classified as global treatment success.

We hypothesized that:

(i) > 85% of scores of ≥ 4 on the VNS will be classed as ‘success’ on the participant‐reported global treatment success scale;

(ii) < 85% but > 50% of scores of 3 on the VNS will be classed as ‘success’ on the participant‐reported global treatment success scale, so this score can be taken to represent partial treatment success;

(iii) the participant’s age (child < 18 years vs. adult ≥ 18 years) and skin type (lighter skin types I–III and darker skin types IV–VI) will have no influence on the interpretation of VNS scores.

Hypotheses (i) and (ii) were assessed separately for HI‐Light trial participants (who made a ‘live’ assessment of their own vitiligo patches, compared with the baseline image) and the independent PPI panel (who used only digital images), to explore the suitability of the VNS for blinded assessment of digital images as an objective outcome measure in trials.

All planned analyses were conducted and reported in full. Some analyses suggested that a VNS score of 3 was more highly valued by trial participants than previously reported. Therefore, we conducted additional post‐hoc κ analyses, taking VNS scores of ≥ 3 to represent treatment success (in addition to VNS ≥ 4) to further explore the construct validity and interpretability of the VNS. We did this for trial participants and the PPI panel.

To explore other possible factors that might influence VNS scores (e.g. lived experience of treating the vitiligo) and to understand how the scales should be used in future trials, we compared responses on the same instrument given by different assessors: (i) trial participant‐rated VNS vs. PPI panel rated VNS; (ii) trial participant‐rated global treatment success vs. PPI panel global treatment success; and (iii) trial participant‐rated global treatment success vs. clinician‐rated global treatment success.

#### Test–retest reliability

To assess the reliability of an outcome measure, two measurements are needed in a group of people who are all assumed to be stable on the construct to be measured.^10^ Test–retest reliability of the VNS was assessed using a random sample of 100 of the image pairs. Two scores for each of the image pairs were completed by 13 PPI raters, who were purposefully selected to represent a range of ages, sex and ethnicity. A period of several weeks was left between the two assessments to prevent recall of previous responses.

#### Effect of hyperpigmentation on Vitiligo Noticeability Scale scores

Our previous work^8^ suggested that hyperpigmentation around a vitiligo patch might be associated with poorer treatment success as defined by the VNS.

We had intended to explore this further, but only 11 participants had post‐treatment hyperpigmentation around their target patch after 9 months of treatment in the HI‐Light trial, making further statistical analysis inappropriate.

### Statistical analyses

#### Construct validity

We converted the five‐point VNS and the four‐point percentage repigmentation scales into binary measures of treatment success (success = VNS scores ≥ 4; percentage repigmentation ≥ 75%), in order to compare these instruments with the binary global treatment success score (Yes/No).

We estimated the crude agreement and κ statistic for:

(a) success on the participant‐reported VNS vs. participant‐reported global treatment success;

(b) success on the PPI panel‐reported VNS (using digital images) vs. participant‐reported global treatment success; and

(c) success based on clinician‐reported percentage of repigmentation (using digital images) vs. participant‐reported global treatment success.

For the scores given by the PPI panel and clinicians, we combined the scores, allowing for clustering at respondent level, and allowing for multiple raters with bootstrapping to estimate 95% confidence intervals (CIs). We used the response given by the majority of raters on the various scales. For transparency, scores are also presented descriptively for all three panel members/clinicians separately.

COSMIN guidance states that for a scale to be acceptable, correlations between two instruments measuring the same construct should be κ ≥ 0·50, or 75% of the results should be in accordance with a priori hypotheses.^10^ However, in this study we took a κ ≥ 0·4 to show an acceptable (moderate) level of agreement with global treatment success, as global treatment success is itself an unvalidated scale and is likely to assess a related, but different, construct and there was remaining uncertainty over the exact cutoff point for defining ‘treatment success’ using the VNS.

#### Interpretability

Results are presented descriptively. Crude agreement and κ statistic calculated are presented, as above, to assess the agreement between treatment success on the VNS (treatment success ≥ 4 and treatment success ≥ 3) vs. global treatment success for participants of different ages (child < 18 years vs. adult ≥ 18 years) and different skin types (skin types I–II, III–IV, V–VI, I–III and IV–VI).

#### Test–retest reliability

This was measured by calculating the intrarater agreement between the two scores given on the five‐point VNS. We conducted a weighted κ analysis, as recommended in COSMIN Guidance^11^ (perfect match in responses, weight = 1·0; discrepancy of one category, weight = 0·5; discrepancy of two or more categories, weight = 0·0). We calculated individual κ scores and 95% CIs for each rater and combined these in a meta‐analysis using random effects to produce an overall reliability estimate.^13^

### Focus groups and online discussion group

We obtained qualitative insights into how people with vitiligo use the VNS, including acceptability, factors which might influence VNS scores, and to explore the need for baseline digital images when making assessments.

We carried out three face‐to‐face focus groups including 21 people with vitiligo (or their parents/carers), followed by two online discussion groups with seven of the same individuals. The focus group discussions were structured to consider the acceptability and utility of the VNS,^14^, ^15^ specifically exploring the role of photographs and the wording of the VNS (the topic guide is shown in Figure S1; see Supporting Information). Data from the focus groups were analysed following the conventions of framework analysis.^16^, ^17^ Initial matrices were themed ‘Vitiligo Noticeability’ (personal reflections) and ‘Scoring Noticeability’; data were coded by P.L. with J.M.B. validating interpretations. The key findings and recommendations were reviewed by all authors and shared with participants in the online discussion groups for final validation (presentation and structured discussion guide are shown in Figure S2; see Supporting Information).

## Results

We had access to 287 pairs of digital images from the HI‐Light trial for participants with complete data at both baseline and end of treatment (9 months). Demographic characteristics are provided in Table 1. Fourteen image pairs were not used in the PPI panel analyses and 18 in the test–retest analysis (due to one or more of the PPI assessors considering the images to be of insufficient quality to make assessments).

**Table 1:** Demographics of HI‐Light participants whose data were used in the validation analyses

Total sample	Construct validity and interpretability *N* = 287 image pairs	Test–retest *N* = 87 image pairs
Mean age at time of consent, years (SD); range	38 (20·9); 5–84	38 (19); 6–76
Participants in each age banding (years), *n* (%)		
< 18	77 (26·8)	
18–40	66 (23)	
41–64	114 (39·7)	
≥ 65	30 (10·5)	
Sex, *n* (%)		
Male	133 (46·3)	40 (46)
Female	154 (53·7)	47 (54)
Ethnicity, *n* (%)		
White	186 (64·8)	51 (59)
Pakistani	25 (8·7)	6 (7)
Indian	19 (6·6)	8 (9)
Bangladeshi	8 (2·8)	4 (5)
Chinese	2 (0·7)	0 (0)
Other Asian	10 (3·8)	3 (3)
Black African	4 (1·4)	2 (2)
Black Caribbean	4 (1·4)	4 (5)
Mixed race	12 (4·2)	1 (1)
Other	16 (5·6)	5 (6)
Rather not say	1 (0·3)	3 (3)
Skin type, *n* (%)		
I	6 (2)	1 (1·1)
II	49 (17·1)	8 (9·2)
III	111 (38·7)	37 (42·5)
IV	53 (18·5)	12 (13·8)
V	61 (21·3)	23 (26·4)
VI	7 (2·4)	6 (6·9)

### Construct validity

*Hypothesis 1*: positive association of ≥ 0·4 between VNS treatment success and participant‐reported global treatment success at end of treatment. This was supported for the trial participants (κ = 0·41; 95% CI 0·31–0·50), but not for the blinded PPI panel (κ = 0·28; 95% CI 0·19–0·37). (Tables 2 and 3).

**Table 2:** Summary of κ‐values from all planned and post hoc analyses

		Trial participant‐assessed global success (main comparator)	PPI‐assessed global success (blinded assessment)	Clinician‐assessed global success (blinded assessment)
	Assessed by	Image at baseline, real time at 9 months	Images at baseline and 9 months	Images at baseline and 9 months
PPI‐assessed global success	Images at baseline and 9 months	0·36		
Clinician‐assessed global success	Images at baseline and 9 months	0·20		
Participant VNS success (≥ 4)	Image at baseline, real time at 9 months	0·41		
PPI VNS success (≥ 4)	Images at baseline and 9 months	0·28	0·62	
Clinician % repigmentation success (≥ 75%)	Images at baseline and 9 months	0·17		0·80

Post hoc analysis to inform interpretability: participant VNS success (≥ 3) vs. participant‐assessed global success: κ = 0·71.

PPI, Patient and Public Involvement panel; VNS, Vitiligo Noticeability Scale.

**Table 3:** Construct validity of VNS compared with global treatment success

	Global treatment success							
VNS^a^	HI‐Light trial participants *n* (%), *N* = 286^b^		PPI assessments using digital images at baseline and end of treatment *n* (%), *N* = 273^b^ for each					
			Assessor 1		Assessor 2		Assessor 3	
	Yes	No	Yes	No	Yes	No	Yes	No
Not successful								
1	5 (9·3)	49 (90·7)	1 (2·2)	44 (97·8)	0	23 (100)	0	62 (100)
2	13 (14·6)	76 (85·4)	6 (4·9)	117 (95·1)	0	162 (100)	0	99 (100)
Partially successful								
3	63 (75)	21 (25)	34 (72·3)	13 (27·7)	46 (95·8)	2 (4·2)	30 (53·6)	26 (46·4)
Successful								
4	49 (98)	1 (2)	43 (100)	0	29 (100)	0	41 (97·6)	1 (2·4)
5	8 (88·9)	1 (11·1)	15 (100)	0	11 (100)	0	14 (100)	0

^a^ Score and classification; ^b^number of participants’ images varies due to individual quality assessment of the digital images and availability of outcome data.

PPI, Patient and Public Involvement panel; VNS, Vitiligo Noticeability Scale.

*Hypothesis 2*: that VNS scores would have better association with participant‐reported global treatment success than percentage repigmentation. This was supported for trial participants (κ = 0·41; 95% CI 0·31–0·50 vs. κ = 0·17; 95% CI 0·10–0·25) and for the blinded PPI panel (κ = 0·28; 95% CI 0·19–0·37 vs. κ = 0·17; 95% CI 0·10–0·25) (Table 2).

The construct validity of the percentage repigmentation scale when compared with clinician‐rated global success was confirmed (κ = 0·80), but not compared with participant‐reported global success (κ = 0·17) (Tables 2 and 4).

**Table 4:** Construct validity of clinician‐rated percentage repigmentation compared with global treatment success

	Clinician assessments using digital images at baseline and end of treatment Global treatment success, *n* (%),^b^ *N* = 287 for each					
Percentage repigmentation^a^	Assessor 1		Assessor 2		Assessor 3	
	Yes	No	Yes	No	Yes	No
0–24%	12 (5·7)	197 (94·3)	0	191 (100)	0	190 (100)
25–49%	19 (67·9)	9 (32·1)	0	31 (100)	0	28 (100)
50–74%	22 (88)	3 (12)	3 (12·5)	21 (87·5)	2 (6·3)	30 (93·8)
75–100%	23 (92)	2 (8)	40 (97·6)	1 (2·4)	37 (100)	0
Total	76 (26·5)	211 (73·5)	43 (15)	244 (85)	39 (13·6)	248 (86·4)

^a^ < 75% = not successful; ≥ 75% = successful; ^b^% of all percentage repigmentation scores.

Overall κ = 0·80. Bootstrapping produces 95% confidence intervals of 0·70–0·90. Crude agreement = 95·5%.

### Interpretability

The proportion of VNS scores of ≥ 4 classed as ‘success’ on the global treatment success scale was as hypothesized (range 88·9–100% for trial participants and PPI panel assessors) (Table 3). Likewise, the proportion of VNS score ≤ 2 classed as success on the global treatment success scale was low and as predicted (range 0–14·6%).

For the partial treatment response (VNS score 3), the proportion of responses classed as a success was as hypothesized for trial participants (75% of VNS 3 were judged as a treatment success). For the PPI panel this ranged from 53·6% to 95·8% (Table 3).

A post hoc analysis exploring agreement between participant‐reported global treatment success and a VNS score of ≥ 3 showed much better agreement (κ = 0·71) than with a VNS score of ≥ 4 (κ = 0·41; Table 2), suggesting that a cutoff of ≥ 4 for classifying VNS treatment success might underestimate the effectiveness of treatments from a trial participants’ point of view.

Table 5 shows the VNS scores and global treatment success for trial participants, grouped by age (child vs. adult). The participant’s age did not influence VNS scores; the κ for children < 18 years (taking VNS of ≥ 4 to represent treatment success) was 0·40, and for adults ≥ 18 years it was 0·41.

**Table 5:** VNS scores vs. global treatment success for HI‐Light trial participants (at 9 months), grouped by age (child vs. adult)^a^

	Global treatment success			
Participant‐rated VNS	Child < 18 years, *n* (%), *N* = 77		Adult ≥ 18 years, *N* = 209	
	CA 68·8%; κ = 0·40		CA 71·8%; κ = 0·41	
	Yes	No	Yes	No
1	1 (7·1)	13 (92·9)	4 (10)	36 (90)
2	3 (14·3)	18 (85·7)	10 (14·7)	58 (85·3)
3	19 (82·6)	4 (17·4)	44 (72·1)	17 (27·9)
4	16 (100)	0	33 (97·1)	1 (2·9)
5	2 (66·7)	1 (33·3)	6 (100)	0
Total	41 (53·2)	36 (46·8)	97 (46·4)	112 (53·6)

^a^ The participant’s age did not influence VNS scores (success represented by VNS ≥ 4 for both groups).

CA, crude agreement; VNS, Vitiligo Noticeability Scale.

Table 6 shows the VNS scores and global treatment success ratings for participants, grouped by skin type. The participant’s skin type did not influence VNS scores; the κ for different groupings of skin type ranged from 0·35 to 0·45.

**Table 6:** VNS scores vs. global treatment success for HI‐Light trial participants (at 9 months), grouped by skin type^a^

	Global treatment success									
	Skin types I–II, *N* = 54		Skin types III–IV, *N* = 164		Skin types V–VI, *N* = 68		Skin types I–III, *N* = 165		Skin types IV–VI, *N* = 121	
	CA 66·7%; κ = 0·35		CA 72·0%; κ = 0·38		CA 72·1%; κ = 0·47		CA 70·3%; κ = 0·37		CA 71·9%; κ = 0·45	
Participant‐rated VNS	Yes, *n* (%)	No, *n* (%)	Yes, *n* (%)	No, *n* (%)	Yes, *n* (%)	No, *n* (%)	Yes, *n* (%)	No, *n* (%)	Yes, *n* (%)	No, *n* (%)
1	0	10 (100)	5 (13·9)	31 (86·1)	0	8 (100)	5 (13·9)	31 (86·1)	0	18 (100)
2	5 (26·3)	14 (73·7)	5 (9·6)	47 (90·4)	3 (16·7)	15 (83·3)	7 (12·7)	48 (87·3)	6 (17·7)	28 (82·4)
3	12 (92·3)	1 (7·7)	36 (70·6)	15 (29·4)	15 (75)	5 (25)	36 (78·3)	10 (21·7)	27 (71·1)	11 (29)
4	10 (90·9)	1 (9·1)	24 (100)	0	15 (100)	0	25 (96·2)	1 (3·9)	24 (100)	0
5	1 (100)	0	1 (100)	0	6 (85·7)	1 (14·3)	2 (100)	0	6 (85·7)	1 (14·3)
Total	28 (51·9)	26 (48·21)	71 (43·3)	93 (56·7)	39 (57·3)	29 (42·7)	75 (45·4)	90 (54·6)	63 (52·1)	58 (47·9)

CA, crude agreement; VNS, Vitiligo Noticeability Scale.

^a^ The patient’s skin type did not influence VNS scores; all VNS scores were ≥ 4.

Post hoc analyses exploring the agreement between assessors made using the same outcome instrument suggest that trial participants assessed the vitiligo differently to blinded outcome assessors (Tables S1–S3; see Supporting Information). Agreement was slightly better for VNS scores than it was for global success scores.

### Test–retest reliability

κ scores for agreement between repeated scoring of VNS assessments on two occasions ranged from κ = 0·49 to κ = 0·83 for the 13 PPI raters. The agreement for all raters combined was κ = 0·69 (95% CI 0·63–0·74).

### Focus groups and online discussion groups

Key themes and data are shown in Figures S1 and S2. People with vitiligo agreed that ‘noticeability’ is a meaningful concept with respect to their vitiligo, and found the VNS a quick, easy and acceptable scale to use for assessing the cosmetic acceptability of repigmentation:

‘Because it’s such a simple question, you could … you could do it January to December, it’s one question…’ (Focus group 1 discussion)

In the context of clinical trials, they agreed that the current wording of the scale makes it clear that the reference point for comparison is the appearance of the vitiligo before treatment. They stated that it was important to give clear instructions when using the scale; particularly that the VNS relates to treatment response at a specified patch of vitiligo and that ‘noticeability’ is from the perspective of the person with vitiligo, not other people.

Focus group participants felt it would be helpful to use other scales, addressing psychosocial aspects of the vitiligo (such as the Vitiligo Impact Patient scale)^18^ alongside the VNS.

Female 3: ‘I think there are two: “How noticeable is your vitiligo?” I think most people would look at it and go “Yes, it’s this noticeable or that noticeable”. But if there isn’t a follow‐up, which is “How do you feel about it?”, that is equally as important as how it shows.’

Male 2: ‘And I don’t think the two are mutually exclusive…So it’s objective and subjective isn’t it? … The objective is the actual patch; the subjective is my own emotion.’ (Focus group 2 discussion)

Focus group participants also indicated that photographs were helpful when the VNS is used to assess treatment response in a clinical trial. Participants stated that although they were constantly ‘monitoring’ their vitiligo subconsciously, and so could judge some changes without images, they agreed that images helped to reinforce the context and purpose of using the VNS (making a comparison to before treatment) and should therefore be used when available:

‘I take photos every six months, my patches, because I’ve been doing various things to try and improve it … I think you notice yourself, you know, if you look at your hand and think oh that looks a bit different, but without having something to compare it to, which I do now … you can actually see, even if it’s the tiniest little change, you can still compare it to something else, so I’d always recommend to people to take photos of patches if they’ve got vitiligo.’ (Focus group 3)

They emphasized that the quality of the digital images is also important.

## Discussion

This study confirmed the construct validity and reliability of the VNS and confirmed its suitability for use for people with vitiligo of all ages and skin types. Focus group findings supported previous recognition that the VNS is acceptable, easy to use and has content validity.^7^

The interpretation of VNS scores was confirmed as: ‘treatment success’ (VNS 4 or 5); ‘partial treatment response’ (VNS 3); and ‘unsuccessful treatment response’ (VNS 1 or 2). However, for those who gave a score of 3 on the VNS, 75% of the trial participants considered this to be a treatment success, suggesting that ‘partial treatment response’ is likely to be of clinical value to many people. Moreover, a post hoc analysis exploring agreement between participant‐reported global treatment success and a VNS score of ≥ 3 showed much better agreement (κ = 0·71) than with a VNS score of ≥ 4. It is also noteworthy that 14·6% of the trial participants (compared with 1·6% of the blinded PPI panel) valued a VNS score of 2, as this represented a halt in the spread of the vitiligo. This may be because, for the HI‐Light trial, all participants had active vitiligo at baseline. All these observations suggest that trial outcomes should be interpreted within the broader context of patient expectation of benefit (repigmentation or stopping the spread of the actively spreading disease) and that reporting of all five VNS categories is valuable. Focus group participants said that the five‐point structure of the scale was meaningful to them, allowing greater granularity of response, which made scoring easier than with a binary ‘success/nonsuccess’ assessment.

Agreement between assessments made by trial participants and those made by the PPI panel or clinicians was generally low. This suggests that it is important that trial outcomes are assessed by participants themselves rather than blinded observers, because lived experience and context around the expectations of treatment are likely to be important.

Focus group participants stated that the comparative wording of the VNS (‘before treatment’) provided a clear reference point, making it suitable for use within a clinical trial. They agreed that baseline photographs were helpful when making assessments and emphasized the need for clear instruction that the assessments relate to a specific vitiligo patch.

In our previous VNS validation study,^8^ assessments were made by people with vitiligo using before and after digital images to show treatment response. In that study, 89·4% and 99% of the images were classed as a treatment success for VNS scores of 4 and 5, respectively, and 95% and 87% of the images were classed as unsuccessful for VNS scores of 1 and 2, respectively. A score of 3 was considered successful for 65% of the image pairs. In the current study, using assessments made by trial participants, the interpretation of VNS scores was more nuanced and lower VNS scores were still valued highly by trial participants, suggesting that interpretation of the VNS scores is likely to be influenced by the context of the study and treatment expectations.

Our findings confirm the previously reported recommendation that treatment success based on percentage repigmentation should be defined as ≥ 75% repigmentation.^8^, ^19^ Percentage repigmentation (in quartiles), as a measure of treatment success, seems to have good construct validity, although further studies will be needed to validate it fully. Nevertheless, the poor association between percentage repigmentation and trial participant‐reported global success (κ = 0·17) suggests that percentage repigmentation is less meaningful for patients and should be interpreted with caution.

There are some limitations. Some of the authors of this work also developed the VNS,^7^, ^8^ so there is potential for conflict of interest in the interpretation and presentation of these results. We encourage other groups to replicate these findings. We used participant‐reported global treatment success as a comparator ‘gold standard’ outcome for some analyses, but, like the percentage repigmentation scale, it has not been formally validated. Although around 40% of the images used were from participants with skin phototypes IV–VI, studies involving greater proportions of participants with darker skin types may have reached different conclusions.

The HI‐Light trial participants had access to baseline photographs when making VNS assessments; the VNS may not have the same measurement properties if baseline photographs are not available. However, feedback from the focus group participants would suggest that baseline photographs should be made available whenever possible. This work also differs from the initial work to validate the VNS,^8^ which used only pre‐ and post‐treatment images, some of which were digitally manipulated, rather than the ‘live’ assessments which the HI‐Light trial participants made of their own vitiligo patches.

Our analyses included data and images from HI‐Light trial participants who had no missing data and who remained in the trial (287 of the 517 participants recruited). The views of these participants may be different to the views of those who were lost to follow‐up.

Recent survey work^19^, ^20^ suggested that the level of repigmentation considered ‘successful’ may vary according to the anatomical location of the vitiligo, with lower levels of repigmentation being acceptable on nonfacial sites. Our work did not take the anatomical location of patches into account. We would also emphasize that the VNS has been developed and validated for assessment of ‘target’ patches of vitiligo, rather than an overall assessment of multiple patches.

The VNS appears to be a valid and acceptable PROM for assessing ‘cosmetically acceptable repigmentation’ in clinical trials. It can be used regardless of age and skin phototype. Those using the VNS need clear instruction that the scale relates to a single patch rather than all vitiligo patches and we would suggest that baseline photographs should be made available for assessments. All five categories of the VNS should be reported to aid interpretation of trial results and to facilitate meta‐analyses in systematic reviews.

Trial participants, PPI panel members and blinded clinicians all assess treatment response slightly differently. This presents methodological challenges for conducting vitiligo trials. Blinding of participants to treatment allocation is advisable where possible, so that people taking part in the trial can provide blinded outcome assessments for themselves. The lower levels of agreement between trial participants and the PPI panel suggest that there may be a difference between what the participants and the PPI panel considered to be a treatment success. For example, cessation of spread of an active vitiligo patch may be valued by a trial participant and be given a higher treatment success score. However, this may not manifest as a significant change in the appearance of the patch as shown in the digital images, such that a blinded assessor (PPI panel member) may give a lower score.

In the HI‐Light trial, both the VNS and percentage repigmentation scale demonstrated a change from baseline in the same direction. This suggests that the VNS is responsive to change but formal assessment of responsiveness was not possible, as the VNS already incorporates the concept of change over time in the wording of the scale itself.

In conclusion, these findings support the use of the VNS as a feasible, valid and reliable tool for assessing response to treatment in vitiligo trials, in terms of ‘cosmetically acceptable repigmentation’.

## Author contributions

**Jonathan Batchelor:** Conceptualization (equal); data curation (equal); formal analysis (equal); funding acquisition (equal); investigation (equal); methodology (equal); project administration (equal); writing – original draft (equal); writing – review and editing (equal). **Sonia Gran:** Conceptualization (equal); data curation (equal); formal analysis (equal); funding acquisition (equal); investigation (equal); methodology (equal); writing – original draft (equal); writing – review and editing (equal). **Paul Leighton:** Conceptualization (equal); data curation (equal); formal analysis (equal); funding acquisition (equal); investigation (equal); methodology (equal); project administration (equal); writing – original draft (equal); writing – review and editing (equal). **Laura Howells:** Conceptualization (equal); formal analysis (equal); investigation (equal); methodology (equal); writing – original draft (equal); writing – review and editing (equal). **Alan Montgomery:** Conceptualization (equal); formal analysis (equal); funding acquisition (equal); investigation (equal); methodology (equal); writing – original draft (equal); writing – review and editing (equal). **Wei Tan:** Conceptualization (equal); data curation (equal); formal analysis (equal); investigation (equal); methodology (equal); writing – original draft (equal); writing – review and editing (equal). **Isma Ahmed:** Data curation (equal); formal analysis (equal); investigation (equal); writing – original draft (equal); writing – review and editing (equal). **Kim S Thomas:** Conceptualization (equal); formal analysis (equal); funding acquisition (equal); investigation (equal); methodology (equal); writing – original draft (equal); writing – review and editing (equal).

## Funding sources

This study was funded by the National Institute for Health Research (NIHR) Research for Patient Benefit Programme (project reference PB‐PG‐1217‐20026). Data used in analyses were in part derived from the HI‐Light Vitiligo trial, which was funded by the NIHR Health Technology Assessment Programme (project reference 12/24/02). The views expressed are those of the author(s) and not necessarily those of the NIHR or the Department of Health and Social Care.

## Conflicts of interest

All authors’ organizations received financial support from the study funder in order to deliver the submitted work; no authors received any additional support from any organization for the submitted work; no authors reported financial relationships with any organizations that might have an interest in the submitted work in the previous 3 years; no authors reported other relationships or activities that could appear to have influenced the submitted work. J.M.B., A.A.M., W.T. and K.S.T. were all involved in the original development of the VNS.

## Data availability

The data that support the findings of this study are available from the corresponding author upon reasonable request.

## Ethics statement

Ethical approval was not required for secondary analysis of the HI‐Light trial dataset; this used digital image pairs from participants who had given informed consent for their trial data to be used in future research. For the focus groups, approval was obtained from the University of Nottingham Faculty of Medicine and Health Sciences Research Ethics Committee (Ref 387‐1909).

## Supporting information


**Table S1** Participant‐rated VNS vs. PPI panel‐rated VNS.
**Table S2** Participant‐rated global treatment success vs. PPI panel‐rated global treatment success.
**Table S3** Participant‐rated global treatment success vs. clinician‐rated global treatment success.
**Figure S1** Focus groups: format, topic guide and main themes identified.
**Figure S2** The Vitiligo Noticeability Scale (VNS); Online Discussion Group 26 May 2020.
