# Supplementary material for: Using the Vitiligo Noticeability Scale in clinical trials: construct validity, interpretability, reliability and acceptability
Source: Br J Dermatol. 2022 Jul 7;187(4):548–56. doi: 10.1111/bjd.21671 (PMC9796274; doi:10.1111/bjd.21671)
Supplement: Supplementary file 1 — Table S1 Participant‐rated VNS vs. PPI panel‐rated VNS. Table S2 Participant‐rated global treatment success vs. PPI panel‐rated global treatment success. Table S3 Participant‐rated global treatment success vs. clinician‐rated global treatment success. Figure S1 Focus groups: format, topic guide and main themes identified. Figure S2 The Vitiligo Noticeability Scale (VNS); Online Discussion Group 26 May 2020. [file BJD-187-548-s001.docx]

**Supporting Information**

Table S1. Participant-rated VNS vs. PPI panel-rated VNS.

Table S2. Participant-rated global treatment success vs. PPI panel-rated global treatment success.

Table S3. Participant-rated global treatment success vs. clinician-rated global treatment success.

Figure S1. Focus groups: format, topic guide and main themes identified.

Figure S2. The Vitiligo Noticeability Scale (VNS); Online Discussion Group 26 May 2020 (see separate PDF file).

**Table S1. Participant-rated VNS vs. PPI panel-rated VNS**

| **VNS at 9 months** | **PPI Panel Member 1** | | | | | |
| --- | --- | --- | --- | --- | --- | --- |
| **Participant** | **1** | **2** | **3** | **4** | **5** | **Total** |
| **1** | 25 (50) | 18 (36) | 6 (12) | 1 (2) | 0 | 50 |
| **2** | 12 (13.6) | 54 (61.4) | 16 (18.2) | 6 (6.8) | 0 | 88 |
| **3** | 6 (7.5) | 41 (51.3) | 19 (23.8) | 10 (12.5) | 4 (5) | 80 |
| **4** | 2 (4.4) | 10 (21.7) | 6 (13) | 23 (50) | 5 (10.9) | 46 |
| **5** | 0 | 0 | 0 | 3 (37.5) | 5 (62.5) | 8 |
| **Total** | 45 | 123 | 47 | 43 | 14 | 272 |
|  | **PPI Panel Member 2** | | | | | |
| **Participant** | **1** | **2** | **3** | **4** | **5** | **Total** |
| **1** | 13 (26) | 34 (68) | 3 (6) | 0 | 0 | 50 |
| **2** | 5 (5.7) | 67 (76.1) | 12 (13.6) | 3 (3.4) | 1 (1.1) | 88 |
| **3** | 4 (5) | 50 (62.5) | 16 (20) | 7 (8.8) | 3 (3.8) | 80 |
| **4** | 1 (2.2) | 11 (23.9) | 15 (32.6) | 15 (32.6) | 4 (8.7) | 46 |
| **5** | 0 | 0 | 1 (12.5) | 4 (50) | 3 (37.5) | 8 |
| **Total** | 23 | 162 | 47 | 29 | 11 | 272 |
|  | **PPI Panel Member 3** | | | | | |
| **Participant** | **1** | **2** | **3** | **4** | **5** | **Total** |
| **1** | 22 (44) | 19 (38) | 8 (16) | 1 (2) | 0 | 50 |
| **2** | 21 (23.9) | 41 (46.6) | 17 (19.3) | 8 (1.1) | 1 (1.1) | 88 |
| **3** | 15 (18.8) | 30 (37.5) | 22 (27.5) | 11 (13.8) | 2 (2.5) | 80 |
| **4** | 4 (8.7) | 9 (19.6) | 9 (19.6) | 18 (39.1) | 6 (13) | 46 |
| **5** | 0 | 0 | 0 | 3 (37.5) | 5 (62.5) | 8 |
| **Total** | 62 | 99 | 56 | 41 | 14 | 272 |

**Kappa:**

**VNS 1-3/4-5 0.54 (95% CI 0.41, 0.67) Crude agreement 86.0%**

**VNS 1-2/3-5 0.37 (95% CI 0.26, 0.48) Crude agreement 68.8%**

**Table S2. Participant-rated global treatment success vs. PPI panel-rated global treatment success**

| **Participant-rated Global treatment success** | **PPI Panel Member 1** | | **PPI Panel Member 2** | | **PPI Panel Member 3** | |
| --- | --- | --- | --- | --- | --- | --- |
| **Participant** | **Yes** | **No** | **Yes** | **No** | **Yes** | **No** |
| **Yes** | 70 (54.3) | 59 (45.7) | 66 (51.2) | 63 (45.8) | 52 (40.3) | 77 (59.7) |
| **No** | 28 (19.6) | 115 (80.4) | 19 (13.3) | 124 (86.7) | 32 (22.4) | 111 (77.6) |

**Kappa 0.36 (95% CI 0.26, 0.47). Crude agreement 68.8%**

**Table S3. Participant-rated global treatment success vs. clinician-rated global treatment success**

| **Participant-rated Global treatment success** | **Clinician 1** | | **Clinician 2** | | **Clinician 3** | |
| --- | --- | --- | --- | --- | --- | --- |
| **Participant** | **Yes** | **No** | **Yes** | **No** | **Yes** | **No** |
| **Yes** | 54 (39.1) | 84 (60.9) | 37 (26.8) | 101 (73.2) | 30 (21.7) | 108 (78.3) |
| **No** | 22 (14.9) | 126 (85.1) | 6 (4.1) | 142 (96) | 8 (5.4) | 140 (94.6) |

**Kappa 0.20 (95% CI 0.11, 0.28). Crude agreement 60.8%**

**Figure S1 – Focus groups: format, topic guide and main themes identified**

**Format**

**Participants:** HI-Light Trial participants who had agreed to be contacted regarding future research; respondents to emails / social media announcements by The Vitiligo Society and Vitiligo Support UK.

**Facilitators:** Dr Paul Leighton and Dr Jonathan Batchelor. Neither facilitator had met the participants previously.

**Location:** Focus groups were held in Birmingham, London and Nottingham

**Topic Guide**

**Introduction/Briefing**

- Welcome and introduction. Introductions from the 2 research team members conducting focus group (Dr P Leighton and Dr J Batchelor)
- Review of the study and the purpose of the focus group.
- Reminder that participation is voluntary and all information disclosed during the group is confidential and anonymous.

**Discussion topics:**

1. The original VNS measure – share the original VNS with the group and ask them to try it out with images provided.
   1. How easy/intuitive was this.
   2. How useful do people find the VNS.
   3. Now try to use VNS on self (or on recollection of child this morning)
      1. Was that different?
      2. Was that as meaningful?
2. Changing the VNS (i)
   1. How could we make the VNS better?
   2. How useful is a comparison with another, fixed time point?
   3. Does this type of comparison make sense to people?
   4. Are there other types of judgement/comparison that make sense to people?
   5. Suggestions for a new structure?
3. Does current wording rely upon comparing to the past?
   1. Suggestions for new wording?
4. Changing the VNS (ii)
   1. Is a five-point scale meaningful? Would other ways of scoring make more sense?
   2. Would a greater number of categories be meaningful?
   3. How to accommodate (potentially contradictory) improvement in the vitiligo, but increased side effects (such as post-treatment hyperpigmentation)?
5. How to use a new VNS scale?
   1. Score privately (at home)? with a healthcare professional (in a clinical setting)? with their consultant?
   2. Reasons for preference? Difficulties with different settings?
   3. Frequency of use?
   4. Other impact of a VNS scale – empowering/depressing?
6. Anything further? Was there anything further they wanted to add that they did not feel had been discussed so far in the group

**Concluding comment and closing the group:**

- Thanks for their participation
- Reminder of what now would happen to the information gathered and that participation was voluntary

**Six Main Themes identified from Focus Groups**

1) Noticeability is a meaningful concept with regard to my vitiligo.

2) I can judge the noticeability of my vitiligo today without taking a photograph, or looking at a photograph previously taken.

3) The noticeability of my vitiligo might vary at different times of the year.

4) The noticeability of my vitiligo might be affected by how I am feeling.

5) I intuitively recognise changes in my vitiligo and how noticeable it is.

6) To score in a consistent way I need to understand why I am scoring the noticeability of my vitiligo.
